# Osteopontin and its spatiotemporal relationship with glial cells in the striatum of rats treated with mitochondrial toxin 3-nitropropionic acid: possible involvement in phagocytosis

**DOI:** 10.1186/s12974-019-1489-1

**Published:** 2019-05-14

**Authors:** Tae-Ryong Riew, Soojin Kim, Xuyan Jin, Hong Lim Kim, Jeong-Hwa Lee, Mun-Yong Lee

**Affiliations:** 1 Department of Anatomy, Catholic Neuroscience Institute, College of Medicine, The Catholic University of Korea, 222 Banpo-daero, Seoul, 06591 Republic of Korea; 2 Department of Biomedicine and Health Sciences, College of Medicine, The Catholic University of Korea, Seoul, 06591 Republic of Korea; 3 Integrative Research Support Center, Laboratory of Electron Microscope, College of Medicine, The Catholic University of Korea, Seoul, 06591 Republic of Korea; 4 Department of Biochemistry, College of Medicine, The Catholic University of Korea, Seoul, 06591 Republic of Korea; 5 The Institute for Aging and Metabolic Diseases, College of Medicine, The Catholic University of Korea, Seoul, 06591 Republic of Korea

**Keywords:** Osteopontin, 3-NP, Acute brain injury, Astrocyte, Microglia, Glial scar, Phagocytosis

## Abstract

**Background:**

Osteopontin (OPN, SPP1) is upregulated in response to acute brain injury, and based on its immunoreactivity, two distinct forms have been identified: intracellular OPN within brain macrophages and small granular OPN, identified as OPN-coated degenerated neurites. This study investigates the spatiotemporal relationship between punctate OPN deposition and astroglial and microglial reactions elicited by 3-nitropropionic acid (3-NP).

**Methods:**

Male Sprague-Dawley rats were intraperitoneally injected with mitochondrial toxin 3-NP and euthanized at 3, 7, 14, and 28 days. Quantitative and qualitative light and electron microscopic techniques were used to assess the relationship between OPN and glial cells. Statistical significance was determined by Student’s *t* test or a one-way analysis of variance followed by Tukey’s multiple comparisons test.

**Results:**

Punctate OPN-immunoreactive profiles were synthesized and secreted by amoeboid-like brain macrophages in the lesion core, but not by reactive astrocytes and activated microglia with a stellate shape in the peri-lesional area. Punctate OPN accumulation was detected only in the lesion core away from reactive astrocytes in the peri-lesional area at day 3, but had direct contact with, and even overlapped with astroglial processes at day 7. The distance between the OPN-positive area and the astrocytic scar significantly decreased from days 3 to 7. By days 14 and 28 post-lesion, when the glial scar was fully formed, punctate OPN distribution mostly overlapped with the astrocytic scar. Three-dimensional reconstructions and quantitative image analysis revealed numerous granular OPN puncta inside the cytoplasm of reactive astrocytes and brain macrophages. Reactive astrocytes showed prominent expression of the lysosomal marker lysosomal-associated membrane protein 1, and ultrastructural analysis confirmed OPN-coated degenerating neurites inside astrocytes, suggesting the phagocytosis of OPN puncta by reactive astrocytes after injury.

**Conclusions:**

Punctate OPN-immunoreactive profiles corresponded to OPN-coated degenerated neurites, which were closely associated with, or completely engulfed by, the reactive astrocytes forming the astroglial scar in 3-NP lesioned striatum, suggesting that OPN may cause astrocytes to migrate towards these degenerated neurites in the lesion core to establish physical contact with, and possibly, to phagocytose them. Our results provide novel insights essential to understanding the recovery and repair of the central nervous system tissue.

**Electronic supplementary material:**

The online version of this article (10.1186/s12974-019-1489-1) contains supplementary material, which is available to authorized users.

## Background

Osteopontin (OPN), also known as secreted phosphoprotein 1 (SPP1), is a multifunctional phosphoglycoprotein containing an adhesive arginine-glycine-aspartate (RGD) sequence [1–3]. The diverse roles of OPN have been elucidated in various central nervous system (CNS) pathologies, including ischemic stroke, traumatic brain injury, Alzheimer’s disease, and experimental autoimmune encephalitis [4–10]. Accumulating evidence suggests that OPN attenuates acute CNS injuries via modulating inflammatory responses and promoting repair processes [11–14]. In addition, OPN acts as a potent regulator of ectopic calcification after brain insult [8–10, 15]. We have recently demonstrated that after acute striatal injury, OPN mediates neurite degeneration, which closely correlates with ectopic calcification processes [16]. These data suggest that OPN is a multifunctional protein linked to a variety of pathophysiological processes.

It is widely accepted that OPN secreted into the extracellular matrix by microglia acts through paracrine and autocrine signaling, thereby activating microglia and recruiting microglia, macrophages, and astrocytes in the insulted brain [4, 12, 17–21]. OPN drives microglial polarization towards the M2 phenotype, thereby modulating inflammatory responses [22], and attenuates secondary neurodegeneration in the thalamus via attenuation of microglial activation after ischemic stroke [12, 23]. In addition, OPN binds to degenerating neurites, thereby facilitating OPN-mediated phagocytosis in the ischemic brain [8, 10], and has an essential role in macrophage-mediated amyloid-β protein phagocytosis in Alzheimer’s model [24]. Although most studies investigating OPN have focused on microglia/macrophages, there is increasing evidence suggesting the association of OPN with astroglial reactions in the lesioned brain. Upregulation of OPN in reactive astrocytes after different types of brain insults has been previously described [9, 20, 25, 26]. Interestingly, recent studies have shown that macrophage-secreted OPN induces the polarization of reactive astrocytes after stroke [14] and that OPN is essential for astrocyte activation in an injured mouse brain, as well as in the primary culture of astrocytes [27]. In addition, OPN is necessary for the cell adhesion, migration, and survival of retinal astrocytes [28] and regulates metabolic activity in cultured optic nerve head astrocytes [29]. Thus, OPN plays a functional role not only in microglia/macrophages, but also in the astroglial reaction elicited by CNS insults. However, because the dynamics of reactive astrocyte responses over the time course of injury and repair are complex, comprehensive investigation of the temporal interaction between OPN and astrocytes is needed.

The present study was designed to elucidate the detailed spatiotemporal relationship between OPN and reactive astrocytes in a 3-nitropropionic-acid (3-NP)-induced striatal injury model over a 4-week survival period. The mitochondrial toxin, 3-NP, irreversibly inhibits the mitochondrial respiratory chain complex II and impairs mitochondrial energy production [30, 31]. This toxin selectively damages medium-spiny striatal neurons and astrocytes in the well-demarcated lesion core, which triggers astroglial hypertrophy and the resultant astroglial scar formation in the peri-lesional area [16, 32–35]. In our previous study using the same model, we showed that the OPN protein is first localized within dendritic mitochondria, and then accumulates on the surface of degenerating dendrites during the first 2 weeks after 3-NP injection [16]. In this study, we focused our attention on the close spatial relationship between OPN and the reactive astrocytes forming the astroglial scar at later time points (14–28 days) post-lesion, when the glial scar was fully formed using confocal microscopy, ultrastructural analysis employing immuno-electron microscopy (immuno-EM), and correlative light-and-electron microscopy. The results of this study provide novel insight into the role of OPN in glial scar formation and astrocytic phagocytosis following CNS injury that is vital to understanding the recovery and repair of CNS tissue.

## Methods

### Animal preparation

All procedures and provisions for animal care were in accordance with the Laboratory Animals Welfare Act, the Guide for the Care and Use of Laboratory Animals, and the Guidelines and Policies for Rodent Survival Surgery provided by the IACUC (Institutional Animal Care and Use Committee) at the College of Medicine of The Catholic University of Korea (Approval number CUMS-2017-0321-05). IACUC and the Department of Laboratory Animals (DOLA) in the Catholic University of Korea, Songeui Campus accredited the Korea Excellence Animal Laboratory Facility from the Korea Food and Drug Administration in 2017 and acquired full Association for Assessment and Accreditation of Laboratory Animal Care (AAALAC) International accreditation in 2018. All efforts were made to minimize animal suffering and to reduce the number of animals used.

Thirty-two adult male Sprague-Dawley rats (250–300 g, OrientBio, Seongnam, Republic of Korea) were used in this study. The animals were housed in groups of three per cage in a controlled environment at a constant temperature (22 ± 5 °C) and humidity (50 ± 10%) with food (gamma ray-sterilized diet) and water (autoclaved tap water) available ad libitum. They were maintained on a 12-hour light/dark cycle. 3-NP (Sigma-Aldrich, St. Louis, MO, USA) was dissolved in buffered saline (pH = 7.0) and administered intraperitoneally (i.p.) at a dose of 15 mg/kg once daily for 3 days. All 3-NP-injected rats were evaluated daily for the presence of behavioral deficits, and only rats exhibiting neurological deficits, such as hind limb impairment or kyphotic posture, recumbency, or impaired postural adjustments, were included in the experimental group [36].

Animals were euthanized 3, 7, 14, and 28 days after the final injection of 3-NP (*n* = 6 rats for each time point). The control group (*n* = 3) received intraperitoneal injections of the same volume of normal saline for 3 consecutive days. The rats in this group were euthanized 3 days after the final injection. The animals were transcardially perfused with 4% paraformaldehyde in 0.1 M phosphate buffer (PB, pH 7.4) after anesthesia using 10% chloral hydrate (4 mL/kg i.p.). The brain tissues were equilibrated with 30% sucrose in 0.1 M PB and frozen and stored at − 70 °C for light microscopic study.

### In situ hybridization and fluorescence immunohistochemistry

In situ hybridization was performed using antisense and sense riboprobes for *Opn* (GenBank accession number M14656, nucleotides 426–991). Antisense and sense riboprobes synthesized from pBluescript II SK (+) vector (Promega Co., Madison, WI, USA) were labeled with digoxigenin (DIG) using in vitro transcription (DIG RNA Labeling Kit, Roche, Basel, Switzerland), as previously described [8, 20].

Coronal cryostat sections (25-μm thick) were hybridized with antisense or sense probes diluted in hybridization solution (500 ng/mL) at 53 °C for 18 h. Hybridized sections were washed and incubated with biotin-conjugated anti-digoxin (anti-DIG) antibodies (1:200; Jackson ImmunoResearch, Westgrove, PA, USA) overnight at 4 °C, then visualized using Cy3-conjugated streptavidin (Jackson ImmunoResearch). For triple staining, immunohistochemistry analysis was performed following in situ hybridization.

For fluorescence immunohistochemistry, the sections were blocked in blocking buffer (10% normal serum, 1% bovine serum albumin, and 0.1% triton) then incubated at 4 °C overnight with a mix of mouse monoclonal antibody to OPN (1:300; American Research Products, Waltham MA, USA, 01-20002), goat polyclonal antibody to OPN (1:1000; R&D systems, Minneapolis, MN, USA, AF808), rabbit polyclonal antibody to ionized calcium-binding adaptor molecule 1 (Iba1; 1:500; Wako Pure Chemical Co., Osaka, Japan, 019-19741), chicken polyclonal antibody to glial fibrillary acidic protein (GFAP; 1:500; Millipore, Burlington, MA, USA, AB5541), rabbit polyclonal antibody to S100β (1:500; Abcam, Cambridge, UK, ab52642), and rabbit polyclonal antibody to lysosomal-associated membrane protein 1 (LAMP1; 1:200; Abcam, ab24170). Antibody staining was visualized using Cy3-conjugated donkey anti-goat antibody (1:2000; Jackson ImmunoResearch) and Alexa 488/647 goat anti-mouse/rabbit/chicken antibody (1:300; Thermo Fisher, Waltham, MA, USA). Negative staining controls for triple-immunofluorescence involved omission of the primary or secondary antibodies. Counterstaining of cell nuclei was carried out using DAPI (4′,6-diamidino-2′-phenylindole, 1:2000; Roche) for 10 min. Slides were viewed with a confocal microscope (LSM800 with Airyscan; Carl Zeiss Co. Ltd., Oberkochen, Germany) equipped with four lasers (Diode 405, Argon 488, HeNe 543, HeNe 633). Images were converted to TIFF format, and contrast levels were adjusted using Adobe Photoshop v.13.0.

### Immunoelectron microscopy

For pre-embedding immunoelectron microscopy, floating vibratome sections (50-μm thick) from experimental rats at 28 days after 3-NP injection were immunostained with a mouse monoclonal anti-rat OPN antibody. After fixation, dehydration, and embedding in Epon 812 (Polysciences, Warrington, PA, USA), areas of interest were excised and glued onto resin blocks. Ultrathin sections (70-nm thick) were cut and observed under an electron microscope (JEM 1010, JEOL, Tokyo, Japan) with uranyl acetate staining.

For the correlative light- and electron-microscopic study, vibratome sections were cryoprotected with 2.3 M sucrose in 0.1 M PB and frozen in liquid nitrogen. Semi-thin cryosections (2-μm thick) were cut at − 100 °C with a glass knife in a Leica EM UC7 ultramicrotome equipped with an FC7 cryochamber (Leica). The sections were double-labeled at 4 °C overnight using a mix of rabbit polyclonal antibody against Iba1 (1:500; Wako), and mouse monoclonal antibody against OPN (1:150; American Research Products). Antibody staining was visualized using Alexa 488 goat anti-rabbit F(ab′) (1:300; Invitrogen) and Alexa 594-FluoroNanogold goat anti-mouse antibody (1:100, Nanoprobes Inc., Yaphank, NY, USA). Sections were counterstained with DAPI for 10 min. Coverslipped sections were examined with a confocal microscope and photographed at × 200, × 400, or × 630 magnifications with a differential interference contrast setting to find specific areas for later examination by electron microscopy. After the coverslips were floated off the sections, silver enhancement was performed using the HQ silver enhancement kit (Nanoprobes) for 3 min, and the tissues were prepared for electron microscopy as described previously [37, 38].

### Quantitative image analysis and three-dimensional reconstruction

Intensity profiles and assessment of the distance between OPN-positive puncta and astroglial reactions at 3 and 7 days post-lesion and the overlapping area at 7, 14, and 28 days were obtained using Zen 2.3 blue edition (Carl Zeiss Co. Ltd.). Sections taken from the invariable region 0.20–1.20 mm anterior to the bregma [39] were double-labeled with OPN and GFAP at 3, 7, 14, and 28 days (*n* = 5–6 rats per time point). To analyze the distance at 3 and 7 days, three to four areas (320 × 320 μm per field) were selected from each section and the average of five maximum horizontal distances between the lateral borders of the OPN-positive area, and the astroglial scar border abutting the lesion core from the confocal *Z*-stacked images was estimated. To determine the overlapping area covered by OPN puncta within the astroglial scar, three to four areas (270 × 270 μm per field) were selected from each section, and astroglial scar areas and OPN-covered areas within the astroglial scar were measured and compared. Astroglial scar areas were determined by the dense compact reactive astrocytes and their processes.

For three-dimensional (3D) reconstruction, S100β, GFAP, Iba1, and LAMP1 signals were 3D-rendered using IMARIS (Bitplane). To analyze the intracytoplasmic localization of OPN puncta within astrocytes and microglia, 3D-rendered images of S100β or GFAP and Iba1 signals were used to represent astrocytes and microglia, respectively. OPN signals outside of each cell were subtracted to reveal only the co-localized signals using mask properties as previously described [40]. Spot detection was performed to count the intracytoplasmic OPN puncta.

### Statistics

Statistical significance was determined by Student’s *t* test or one-way analysis of variance (ANOVA) followed by Tukey’s multiple comparisons test. Differences with *P* values of less than 0.05 were considered statistically significant. The number of animals and imaging fields used are indicated in the figure legends. All statistical calculations were performed using GraphPad Prism version 5 (GraphPad Software Inc., San Diego, CA, USA). The exact *P* values are indicated in the graphs and the text.

## Results

For triple immunofluorescence, we used one of two kinds of antibodies against OPN, as needed. Double labeling revealed that these two antibodies shared an overlapping expression in the striatum of 3-NP injected rats on day 7, where OPN-positive staining was visible as small granular puncta (Additional file 1: Figure S1).

### Characterization of puncta-like OPN staining in the lesioned striatum

**Fig. 1:**
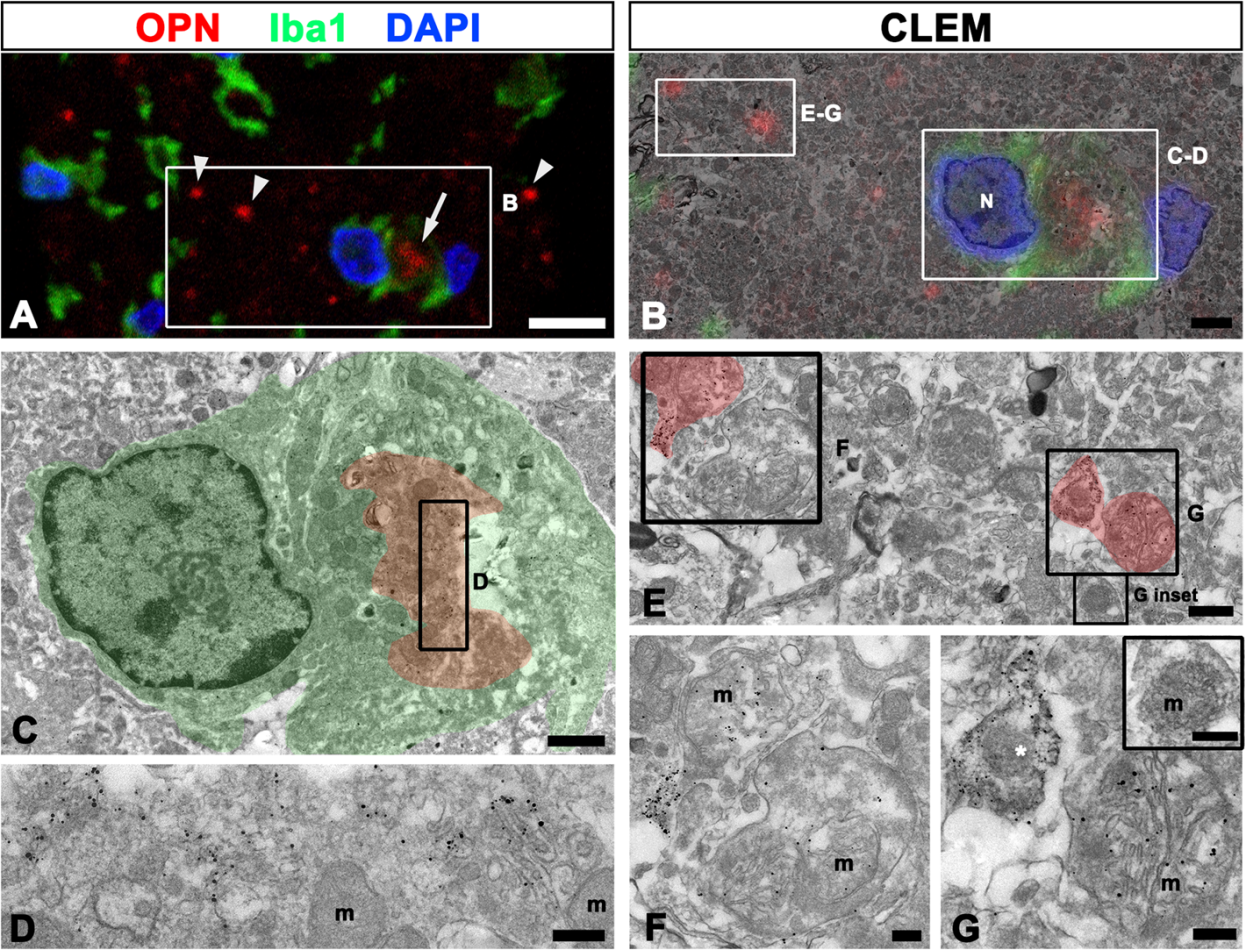
Ultrastructural characterization of OPN in the lesion core 3 days after lesion induction by 3-nitropropionic-acid. Confocal microscopic image of a semi-thin section double-labeled with OPN and Iba1 (**a**), images of confocal data overlaid onto the corresponding electron microscopic image (**b**), and the corresponding transmission electron microscopic images obtained from the same field (**c**–**g**). **a**, **b** Confocal data shows the two different OPN-positive profiles based on their localization and shape: puncta scattered among the activated microglia/macrophages (arrowheads), and puncta localized to the perinuclear region of brain macrophages (arrow). **c**–**d** Higher-magnification views of the boxed areas in **a**. Note that silver-enhanced immunogold particles are specifically localized to the saccules and tubules in the Golgi complex, but not in the mitochondria (m). N, nucleus of an Iba1-positive brain macrophage. **e**–**g** Higher-magnification views of the boxed areas in **b** and **e**, respectively. Note that the OPN-positive puncta are indeed the swollen mitochondria (m in **f** and **g**) with disorganized cristae in the neurites or OPN-coated degenerating neurites containing small and highly electron-dense mitochondria (asterisk in **g**). Also, note that adjacent unlabeled neurites contain mitochondria that appear normal (m in inset of **g**). Cell nuclei appear blue after DAPI staining. Scale bars = 2 μm for **a**, **b**; 1 μm for **c**; 0.2 μm for **d**, **f**, **g** and **g** inset; and 0.5 μm for **e**

Consistent with our previous data [16], OPN-positive staining in the striatum of 3-NP injected rats was visible by light microscopy as small granular puncta, which could be divided into two distinct forms based on their localization: puncta scattered among the Iba1-positive activated microglia/macrophages and puncta localized to the perinuclear region of brain macrophages (Fig. 1a). We first performed correlative light- and immuno-electron microscopic imaging to precisely characterize these OPN puncta. Overlay of the confocal microscopy and transmission electron microscopy images confirmed that intracellular OPN puncta in brain macrophages corresponded to the Golgi complex, in which the silver-enhanced immunogold particles were specifically localized to the saccules and tubules (Fig. 1b–d). In contrast, OPN-positive puncta were indeed swollen mitochondria with disorganized cristae in the neurites (Fig. 1b, e–g) or OPN-coated degenerating neurites containing small and highly electron-dense mitochondria (Fig. 1b, e, g); OPN was not associated with the apparently normal mitochondria (inset in Fig. 1g).

### Cellular localization of OPN mRNA and protein in the lesioned striatum

**Fig. 2:**
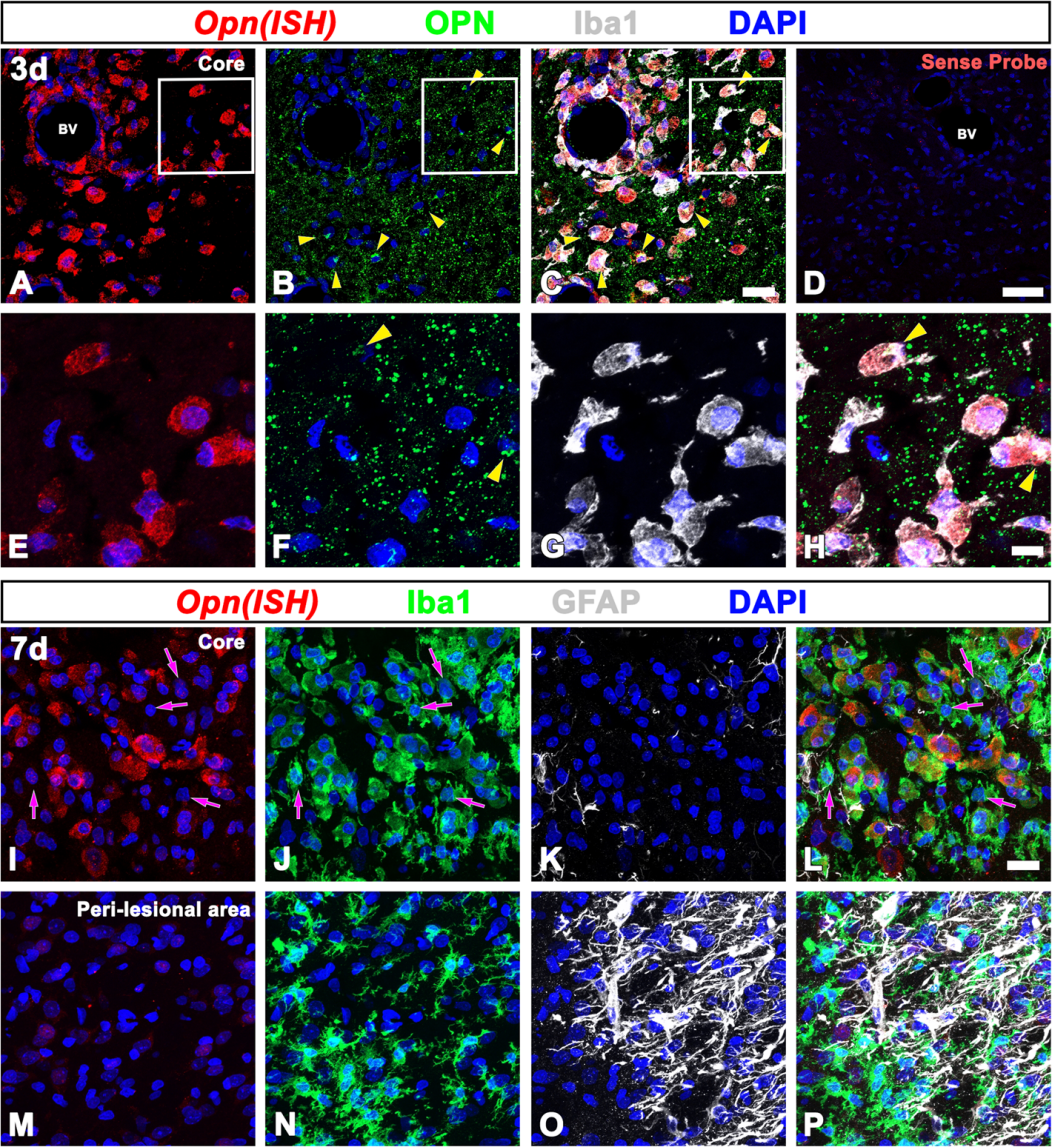
Spatial relationships of *Opn* mRNA and protein in the lesion core in the 3-NP-injured striatum. **a**–**h** Triple labeling for *Opn* mRNA, OPN protein, and Iba1 on day 3 post-lesion. The corresponding boxed areas in **a**–**c** are enlarged in **e**–**h**, respectively. *Opn* mRNA is expressed in Iba1-positive amoeboid-like brain macrophages, while punctate OPN-immunoreactive profiles are visible among the brain macrophages. Note the prominent OPN staining within the cytoplasm of *Opn* mRNA-expressing brain macrophages, and possible localization to the Golgi complex (arrowheads in **b**, **c**, **f**, and **h**). **d** Sections hybridized to a sense-stranded probe showed the specificity of in situ hybridization histochemistry. **i**–**l** Triple labeling for *Opn* mRNA, Iba1, and GFAP on day 7. In the lesion core, *Opn* mRNA is expressed in amoeboid-like brain macrophages but is weak or negligible in activated stellate microglial cells with thick and short processes (arrows in **i**, **j**, and **l**). **m**–**p** In the peri-lesional area, no significant labeling for *Opn* mRNA is observed in either reactive astrocytes or activated microglia with a stellate shape. Cell nuclei appear blue after DAPI staining. Scale bars = 20 μm for **a**–**c** and **i**–**p**, 50 μm for **d**, and 10 μm for **e**–**h**

We next defined the precise localization of, and relationship between, the *Opn* mRNA and the OPN protein in rat striata subjected to 3-NP by triple labeling using in situ hybridization and immunofluorescence. *Opn* mRNA was expressed in Iba1-positive cells, which exhibited amoeboid morphological characteristics, but the expression was weak or negligible in activated stellate microglial cells with thick and short processes on days 3 and 7 post-lesion (Fig. 2a–c, e–l). Thus, *Opn* mRNA expression was induced in brain macrophages, consistent with our previous data [8, 10]. These *Opn*-expressing brain macrophages could have been derived either from resident microglia or from infiltrating blood-borne macrophages, as the two are indistinguishable by morphological criteria due to a lack of specific discrimination criteria [41–43]. Additionally, blood-brain barrier breakdown in the striatum has been reported in rat models of Huntington’s disease induced by 3-NP [35, 44, 45]. As noted above, some OPN-positive puncta were observed within the cytoplasm of *Opn* mRNA-expressing brain macrophages, corresponding to the Golgi complex, but most OPN-immunoreactive puncta were not associated with brain macrophages (Fig. 2e–h). These data confirmed that the OPN protein was synthesized and secreted by brain macrophages in the lesioned striatum. However, no significant labeling for *Opn* mRNA was observed in either reactive astrocytes or activated microglia with a stellate shape in the peri-lesional area (Fig. 2m–p). Specificity for in situ hybridization was verified by the lack of signals when hybridization was carried out in the presence of a sense-stranded probe (Fig. 2d).

### Spatiotemporal relationships among punctate OPN, astrocytes, and microglia in the striatum in the early phase following 3-NP injection

**Fig. 3:**
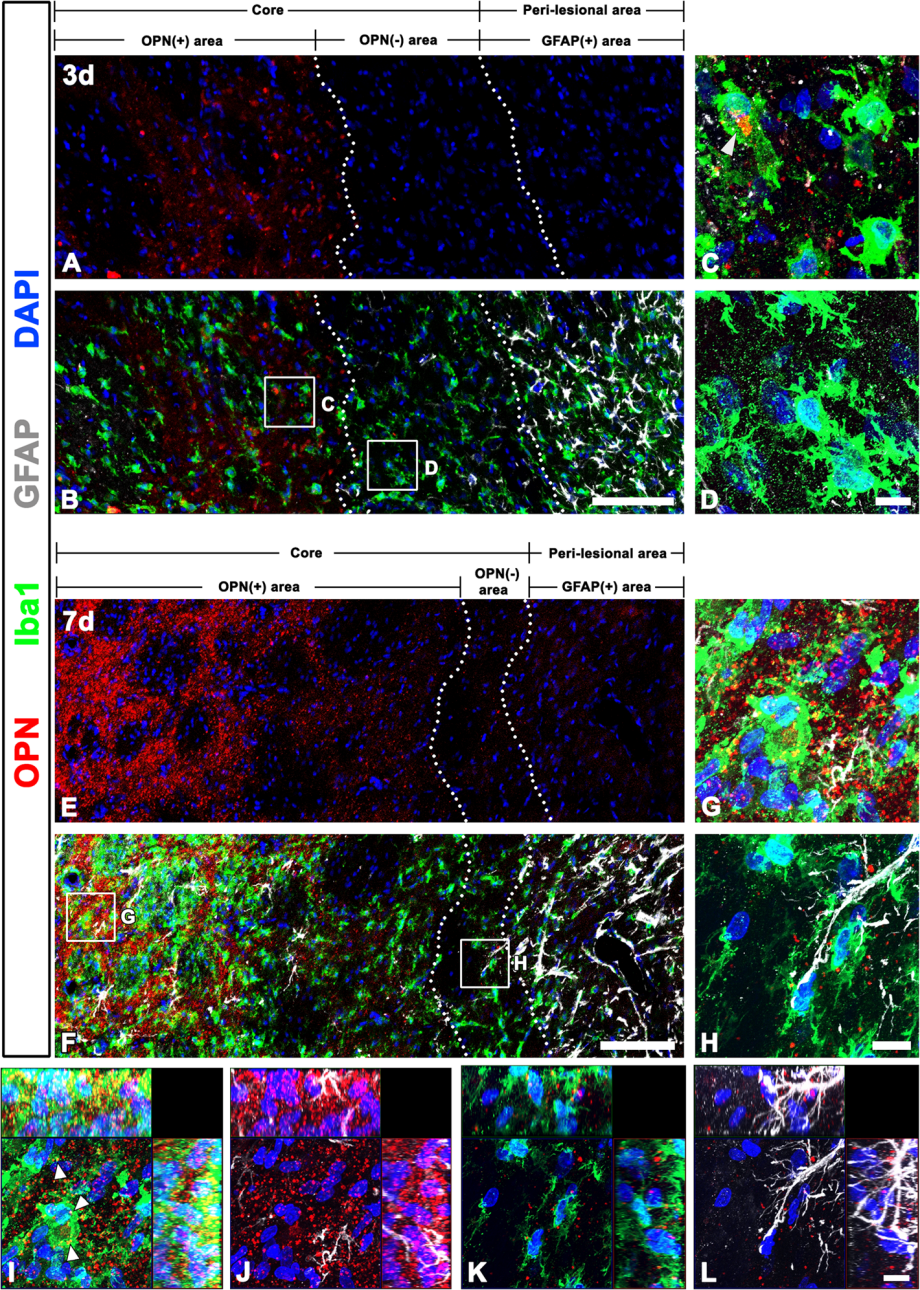
Punctate OPN-immunoreactive profiles and two glial cells 3 and 7 days after 3-NP injection. **a**–**d** Triple labeling with OPN, GFAP, and Iba1 at day 3 post-lesion showing that OPN puncta are observed only in the central lesion core, but not in the periphery of the lesion core, or the peri-lesional area. The two broken lines indicate the borders of the three areas: lesion center, lesion periphery, and the peri-lesional area. The corresponding boxed areas in the lesion center and periphery in **b** are enlarged in **c** and **d**. Note that OPN expression is localized within the Golgi complex (arrowhead) of brain macrophages and among the brain macrophages in the lesion center, while activated microglia with clearly demarcated processes are mainly observed at the OPN-negative lesion periphery. **e**–**h** Triple labeling with OPN, GFAP, and Iba1 at day 7 post-lesion showing that OPN and Iba1 have overlapping regional distribution in the lesion core, despite preferentially localized OPN puncta in the central lesion core. The boxed areas in the lesion center and lesion periphery are enlarged in **g** and **h**. Note that OPN puncta are detectable at the lesion periphery immediately adjacent to the peri-lesional area, in which astroglial hypertrophy and resultant scar formation are evident. **i**–**l** Orthogonal magnified views of **g** and **h** showing the relationship between OPN and glial cells in the lesion center (**i**, **j**) and lesion periphery (**k**, **l**). In the lesion center, OPN puncta are mostly scattered around brain macrophages and some are within their cytoplasm (arrowheads in **i**), while they do not show colocalization pattern with astroglial fibers. In the lesion edge, OPN puncta are sparse and scattered among the activated microglia with evident processes or astroglial fibers but are not localized within both glial cells. Cell nuclei appear blue after DAPI staining. Scale bars = 100 μm for **a**, **b**, **e**, and **f**; 10 μm for **c**, **d**, **g**, **h**, and **i**–**l**

To assess punctate OPN and its spatiotemporal relationship with astrocytes and microglia in the striatal lesion, we performed triple labeling with OPN and two glia-specific markers, GFAP and Iba1 (Fig. 3). On day 3 post-lesion, a well-demarcated lesion core was evident in the lateral part of the striatum, in which GFAP immunoreactivity had virtually disappeared (Fig. 3b), as reported previously [16, 37]. At this time point, OPN-positive puncta were observed only in the central part of lesion core, but neither in the edge of the lesion core nor in the peri-lesional area (Fig. 3a and b). In the OPN-positive lesion core, where amoeboid-like brain macrophages predominated, the puncta were either localized in the Golgi complex of brain macrophages or scattered among the brain macrophages (Fig. 3c). In contrast, activated microglia with clearly demarcated processes were mainly observed at the OPN-negative lesion edge (Fig. 3d). At 7 days post-lesion, there was a notable increase in the number of OPN-positive puncta in the lesion core, where OPN and Iba1 had an overlapping regional distribution (Fig. 3e–h). The relationship between OPN and glial cells was further examined with orthogonal magnified views. In the central lesion core, where only some astroglial fibers were observed, OPN puncta were mostly scattered around brain macrophages, but some appeared to be within their cytoplasm (Fig. 3i and j). In the lesion edge, however, OPN puncta were sparse and scattered among activated microglia with evident processes or some astroglial fibers but were not localized within both glial cells (Fig. 3k, l). Although OPN puncta were still preferentially localized in the central lesion core, they were detectable at the lesion edge immediately adjacent to the peri-lesional area, where astroglial hypertrophy and resultant scar formation were evident.

These findings indicated that the OPN-positive area and the astroglial scar were in close apposition or in direct contact during the first week post-lesion. This finding was further supported by the intensity profiles of OPN- and GFAP-signals across the lesion core and peri-lesional area at 3 and 7 days post-lesion (Fig. 5a–d). Quantitative comparison of the distance between the lateral border of the OPN-positive lesion core and the astroglial scar border abutting the lesion core at 3 and 7 days post-lesion showed that the OPN-positive area was located approximately 193.14 ± 14.33 μm away from the astroglial scar at 3 days, but both were in close apposition, and even overlapped, at 7 days (mean distance 9.46 ± 2.56 μm; Fig. 5i).

### Spatiotemporal relationships among striatum OPN, astrocytes, and microglia in the late phase following 3-NP injection

**Fig. 4:**
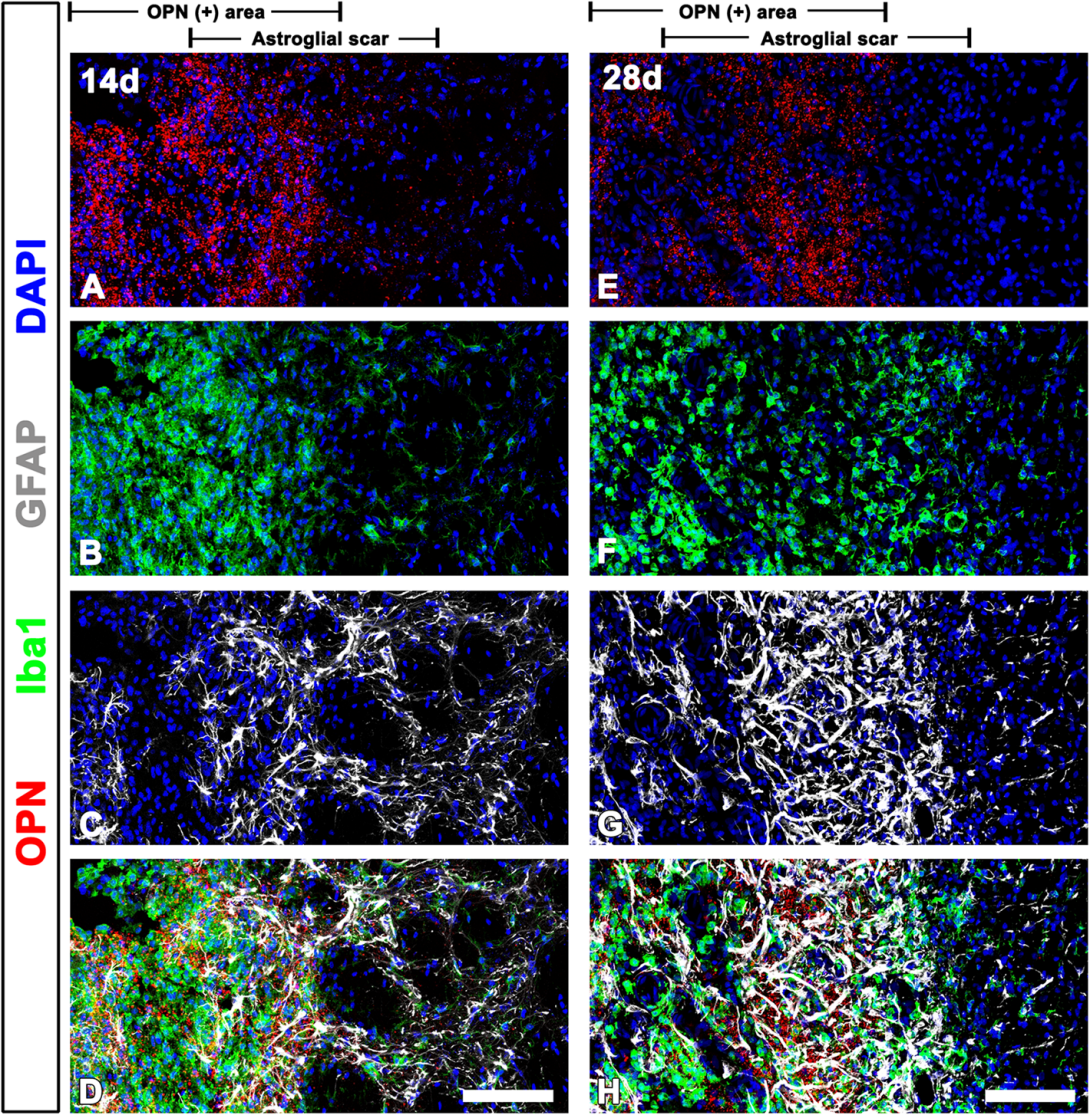
Punctate OPN-immunoreactive profiles and two glial cells 14 and 28 days after 3-NP injection. **a**–**d** Triple labeling with OPN, GFAP, and Iba1 at day 14 post-lesion. Notably, OPN puncta appear to overlap with Iba1 expression in the lesion core but are also detectable within the astroglial scar, predominantly along its border immediately abutting the lesion core. Note that the astroglial scar is demarcated by the presence of hypertrophied and elongated astroglial processes, forming a network. **e**–**h** Triple labeling with OPN, GFAP, and Iba1 at day 28 post-lesion showing that OPN puncta are distributed throughout the astroglial scar, and are particularly abundant in areas forming a prominent, dense, compact scar. Cell nuclei appear blue after DAPI staining. Scale bars = 100 μm for **a**–**h**

**Fig. 5:**
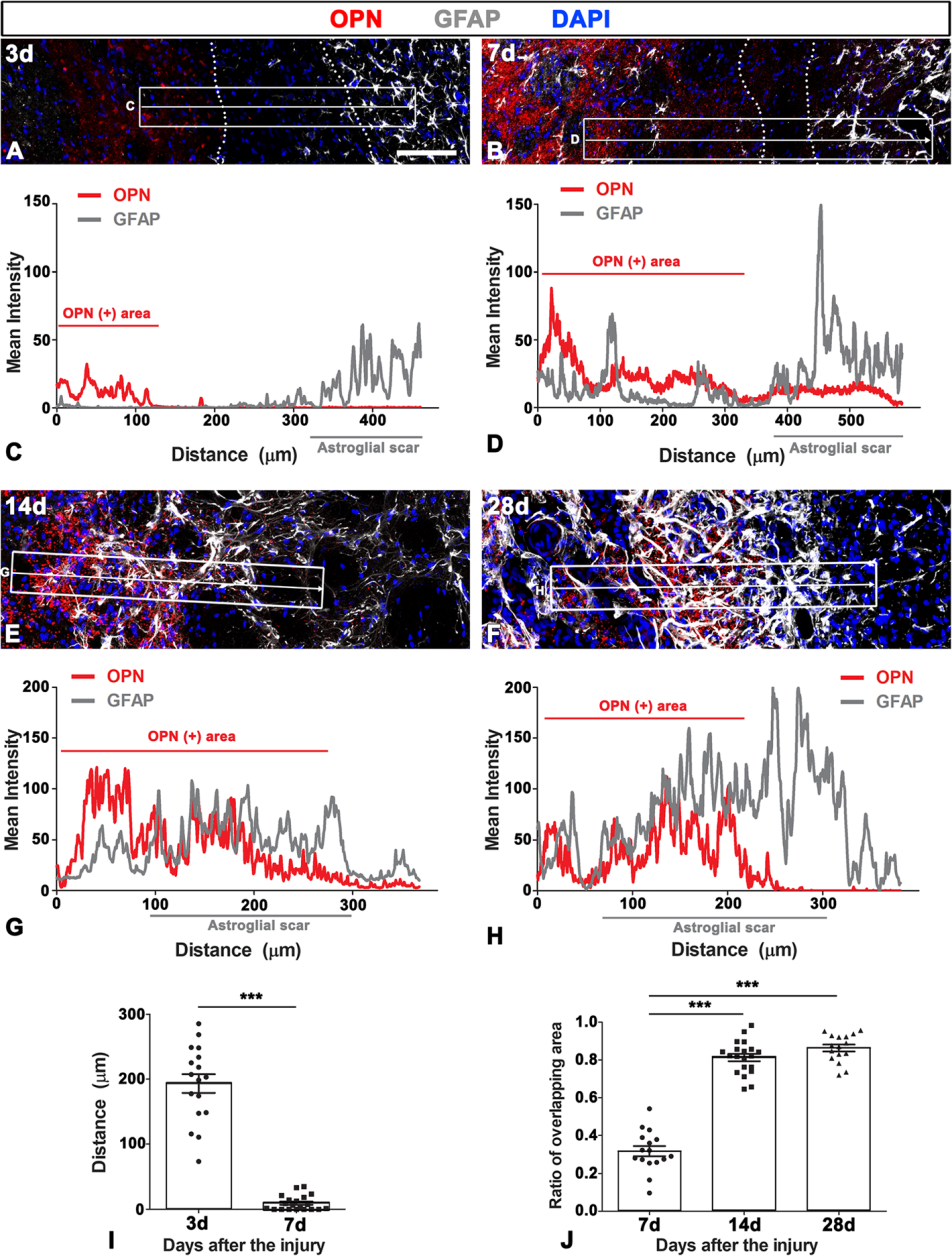
Spatiotemporal relationships of OPN and GFAP in the lesioned striatum. **a**–**h** Fluorescence intensity profiles of OPN and GFAP signals (**c**, **d**, **g**, and **h**) are generated along the indicated area (white arrows in **a**, **b**, **e**, and **f**) from Fig. 3b (3 days post-lesion), Fig. 3f (7 days), Fig. 4d (14 days), and Fig. 4h (28 days), respectively. Note that the OPN signal is localized away from the GFAP signal at 3 days post-lesion but becomes closer and overlaps with GFAP over time. **i** Quantitative analysis of the distance between the lateral border of the OPN-positive area and the astroglial scar border abutting the lesion core at 3 and 7 days post-lesion. Note that both become in close apposition with each other, and even overlap at 7 days (*n* = 17, 20 fields from 5 rats per time point, ^***^P < 0.001 vs. the day 3, unpaired *t* test). **j** Quantitative temporal analysis of the overlapping area covered by OPN and GFAP in the astroglial scar showing that the overlapping area of both signals significantly increases at days 14 and 28 post-lesion (*n* = 16, 20 fields from 6 rats per time point, ^***^P < 0.001 versus the day 7, one-way ANOVA (*P* < 0.0001) with Tukey’s multiple comparison test). Scale bars = 100 μm for **a**, **b**, **e**, and **f**

We next investigated the spatiotemporal relationship among OPN puncta, astrocytes, and microglia in the striatal lesion 14–28 days post-lesion, when the glial scar was fully formed (Fig. 4). Fourteen days post-lesion, OPN puncta still appeared to overlap with Iba1 expression in the lesion core but were also detected within the astroglial scar, predominantly along the border immediately abutting the lesion core (Fig. 4a–d). On day 28, the latest time point examined, OPN-positive puncta could be seen throughout the astroglial scar and were particularly abundant in areas forming a prominent, dense, compact scar (Fig. 4e–h). Fluorescence intensity profiles of OPN and GFAP signals along the astroglial scar at 14 and 28 days post-lesion supported their coincidental distribution pattern (Fig. 5e–h).

To confirm that OPN puncta were indeed associated with reactive astrocytes over time after 3-NP injection, we measured the overlapping area contributed by OPN and GFAP. This analysis revealed that OPN puncta were observed only in a small part (31.68 ± 2.7%) of the astroglial scar at 7 days post-lesion but were detectable within almost all areas of the astroglial scar at days 14 (81.29 ± 1.97%) and 28 (86.3 ± 1.86%) post-lesion (Fig. 5j).

### Intracytoplasmic localization of OPN puncta within astrocytes and microglia in the lesioned striatum

**Fig. 6:**
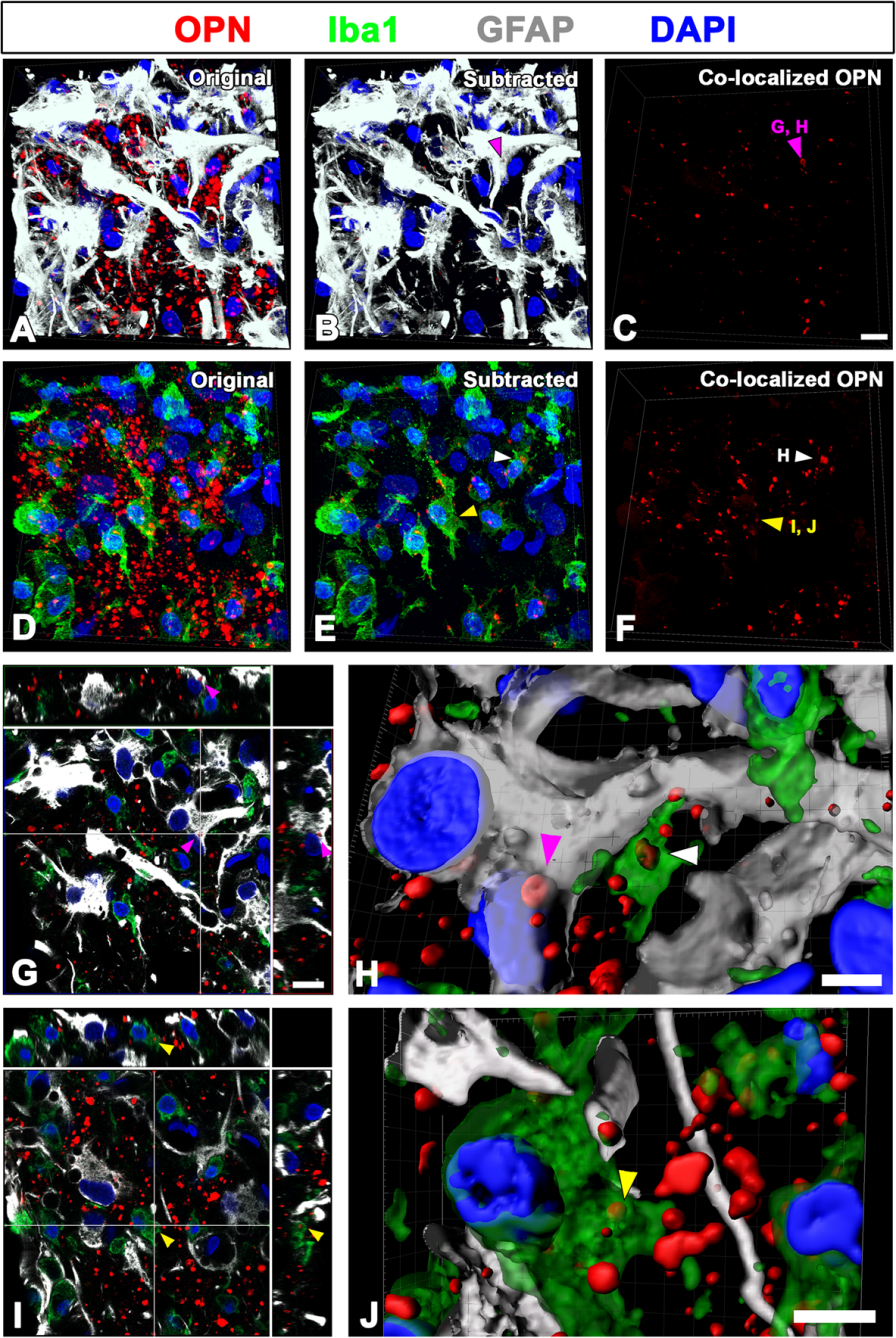
Representative images showing the intracytoplasmic OPN puncta in astrocytes and microglia/macrophages within the astroglial scar. **a**–**f** Three-dimensional views of triple labeling for OPN, Iba1, and GFAP from confocal z-stack images at 28 days post-lesion. **a**, **d** shows the relationship of OPN puncta with reactive astrocytes (**a**) or activated microglia/macrophages (**d**). **b**, **e** Images subtracting the OPN puncta outside glial cells from **a** and **d**, respectively. **c**, **f** Images showing the intracytoplasmic OPN puncta in astrocytes (**c**) and activated microglia/macrophages (**f**). **g**, **i** Orthogonal views showing the presence of OPN puncta within reactive astrocytes (magenta arrowheads in **b**, **c**, **g**, and **h**) or activated microglia/macrophages (yellow arrowheads in **e**, **f**, **i**, and **j**; white arrows in **e**, **f**, and **h**). **h**, **j** The three-dimensional rendering of intracytoplasmic OPN puncta in astrocytes (magenta arrowhead) and activated microglia/macrophages (yellow and white arrowheads). Cell nuclei appear blue after DAPI staining. Scale bars = 10 μm for **a**–**f** and **g**, **i**; 5 μm for **h** and **j**

We next defined the precise localization of and relationship between OPN puncta and two glial cells at 28 days post-lesion. As shown in Fig. 6a–c, reactive astrocytes forming the astroglial scar were frequently observed in close proximity to OPN puncta and occasionally contained such puncta within the cytoplasm. In addition, OPN puncta were localized within the cell bodies of activated microglia/macrophages (Fig. 6d–f). By means of the 3D reconstruction using 3D rendering in IMARIS, we demonstrated that OPN puncta were very closely in contact with the surface of both the astrocytes and the microglia and were also internalized by these two glial cells (Fig. 6g–j).

**Fig. 7:**
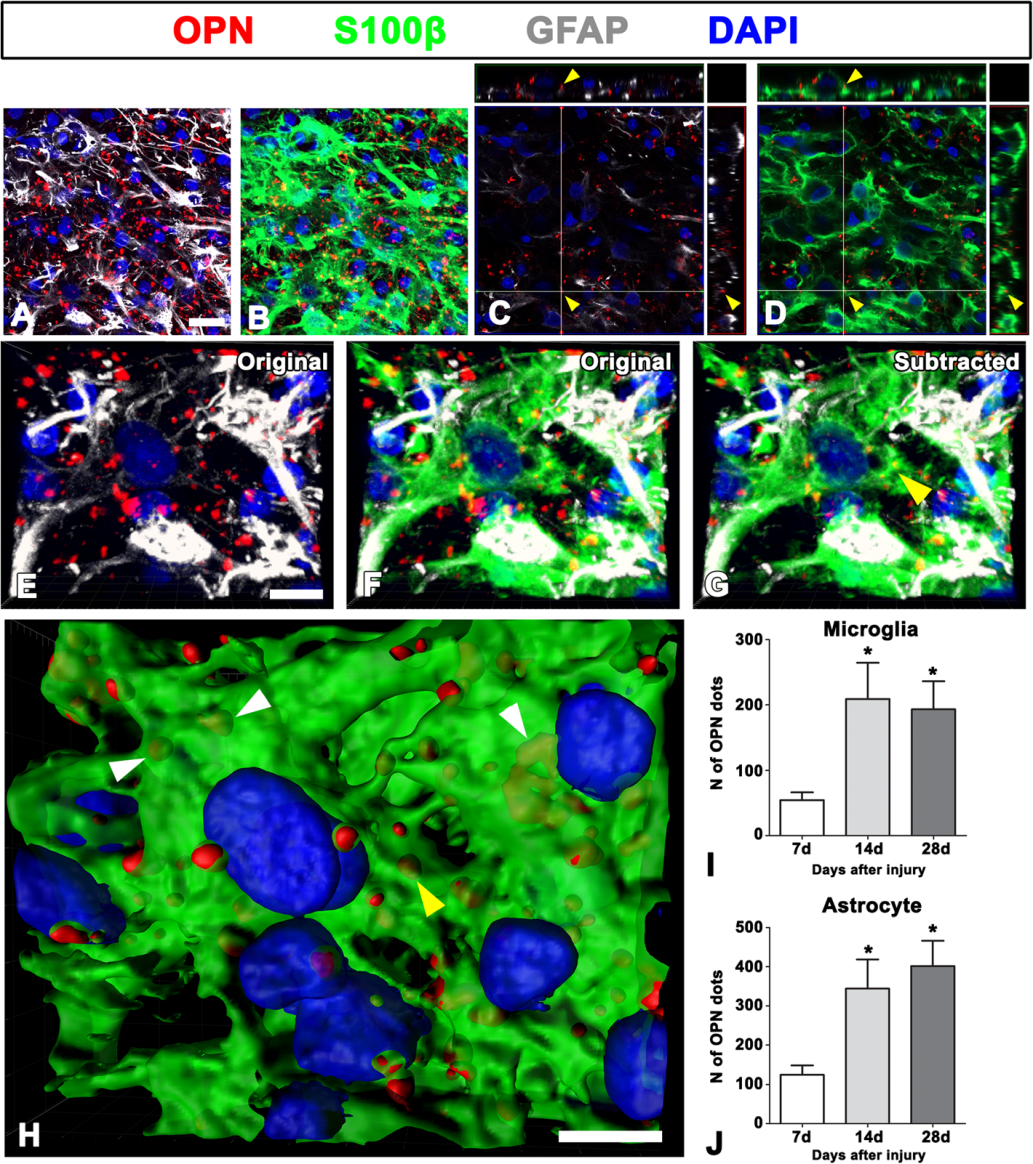
Representative images showing intracytoplasmic OPN puncta in reactive astrocytes forming the astroglial scar. **a**, **b** Triple labeling for OPN, GFAP, and S100β at 28 days post-lesion, showing that GFAP is localized in intermediate filaments, whereas S100β is additionally expressed in the cytoplasm of soma and processes. **c**, **d** Orthogonal views showing that OPN puncta are completely enwrapped by S100β immunoreactivity (arrowheads in **d**), but not by GFAP-immunoreactivity (arrowheads in **d**). **e**–**g** The three-dimensional views from confocal z-stack images of GFAP single-labeled (**e**) and GFAP/S100β double-labeled cells (**f**). **g** An image subtracting the OPN puncta outside astrocytes from **f**, showing that OPN puncta are closely associated with, or completely enwrapped by, reactive astrocytes. Note that GFAP occupies only a small part of the S100β-positive soma and processes in reactive astrocytes. **h** The three-dimensional rendering of the above images showing the intracytoplasmic OPN puncta (arrows) internalized completely by the S100β-positive reactive astrocytes with the hypertrophic cytoplasm. The yellow arrow in **h** indicates the intracytoplasmic OPN puncta shown in **c**, **d**, and **g**. **i**, **j** Quantitative analysis of the time-dependent number of OPN puncta internalized by activated microglia/macrophages (**i**) or astrocytes (**j**) showing that intracytoplasmic OPN puncta in both glial cells significantly increases at days 14 and 28 post-lesion compared with the day 7 data (*n* = 11, 17 fields from 6 rats per time point, ^*^P < 0.05 versus the day 7 one-way ANOVA (*P* = 0.0091 for **j** and *P* = 0.0108 for **i**) with Tukey’s multiple comparison test. Cell nuclei appear blue after DAPI staining. Scale bars = 20 μm for **a**–**d**; 10 μm for **e**–**h**

The presence of intracytoplasmic OPN puncta in reactive astrocytes was substantiated by triple labeling for OPN, GFAP, and the calcium-binding protein S100β (Fig. 7). In reactive astrocytes, GFAP occupied only a small part of the S100β-positive soma and processes (Fig. 7a, b, e, f), which could be attributable to the different expression patterns of the two proteins in the astrocytes; GFAP is localized in intermediate filaments, whereas S100β is additionally expressed in the cytoplasm of soma and processes. As shown in Fig. 7e–h, the 3D rendering of astrocytes forming the astroglial scar revealed that OPN puncta were completely internalized by reactive astrocytes with the hypertrophic cytoplasm.

We next quantified time-dependent changes in the amount of OPN puncta enwrapped by microglia or astrocytes, both of which were located within the glial scar, using 3D confocal reconstruction. Compared to those at 7 days (54.40 ± 11.96), the number of intracytoplasmic OPN puncta in microglia significantly increased at 14 days (208.9 ± 55.55) and 28 days (193.0 ± 43.06) post-lesion, despite the slight decrease of OPN puncta at 28 days (Fig. 7i). In addition, the number of OPN puncta within the cytoplasm of reactive astrocytes significantly increased at 14 (344.2 ± 74.48) and 28 days (401.7 ± 64.51) post-lesion, compared to those at 7 days (123.7 ± 24.06) (Fig. 7j). Interestingly, at each time point, the number of intracytoplasmic OPN puncta of reactive astrocytes was higher than those of the activated microglia/macrophages, yet there were no statistically significant differences.

### Electron micrographs of OPN puncta internalized by reactive astrocytes

**Fig. 8:**
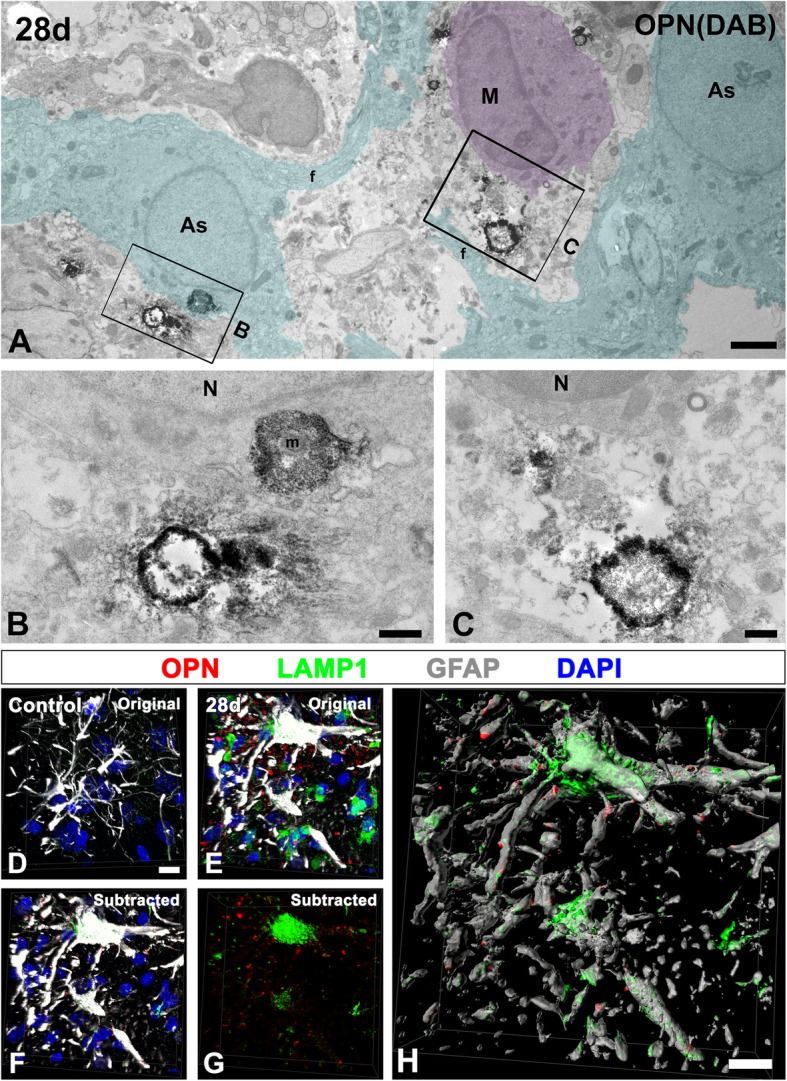
Ultrastructural identification of intracytoplasmic OPN in reactive astrocytes. **a**–**c** Immunoelectron microscopic (EM) images in the lesioned striatum at 28 days post-lesion, showing that degenerated neurites delineated by DAB grains are in close proximity to, or internalized by, reactive astrocytes (As, cyan) showing extensive cell body hypertrophy and cytoplasmic processes. The boxed areas in **a** are enlarged in **b** and **c**. Note that intracytoplasmic OPN-positive profiles are similar in shape to those outside the astrocytes, and occasionally contain small and highly electron-dense mitochondria (m in **b**). The EM image shows representative data for immunoelectron staining of three sections from three rats. As astrocytes, cyan; f glial filaments; M microglia/macrophage, magenta; N nucleus. **d**–**g** Triple labeling with OPN, GFAP, and LAMP1 in control striatum (**d**) and in the lesioned striatum at 28 days post-lesion (**e**–**g**). **e**–**g** Confocal z-stack of images (**e**), an image including only intracytoplasmic OPN and LAMP1 within reactive astrocytes from **e** (**f**) and an image displaying the LAMP1 signals within reactive astrocytes (**g**) showing prominent LAMP1 staining with a typical punctate pattern, is localized within reactive astrocytes that contained OPN puncta. **h** The three-dimensional rendering of reactive astrocytes that contain numerous lysosomes and OPN puncta. Cell nuclei appear blue after DAPI staining. Scale bars = 2 μm for **a**; 0.5 μm for **b**, **c**; 10 μm for **d**–**h**

The presence of intracytoplasmic OPN puncta in reactive astrocytes was further analyzed by immunoelectron microscopy in the lesioned striatum of rats at 28 days post-lesion (Fig. 8). OPN puncta, which were indeed degenerated neurites delineated by DAB grains indicative of OPN, were in close proximity to, and even internalized by, reactive astrocytes showing extensive cell body hypertrophy and cytoplasmic processes (Fig. 8a–c). Such OPN-labeled profiles within reactive astrocytes were similar in shape to those in close contact with the astrocytes and occasionally contained small and highly electron-dense mitochondria, as described above.

To further define whether OPN puncta were phagocytosed by reactive astrocytes, we performed triple labeling for OPN, GFAP, and LAMP1, a late endosomal/lysosomal marker. We found, using confocal microscopy and enhanced visualization by means of 3D-rendering approaches that prominent LAMP1 staining showing a typical punctate pattern was localized within the reactive astrocytes that contained OPN puncta (Fig. 8f–h), while astrocytes of the saline-treated controls contained only a few LAMP1-positive lysosomes (Fig. 8d). In addition, LAMP1 staining was prominent in activated microglia/macrophages in the lesioned striatum of rats at 28 days post-lesion (Additional file 2: Figure S2).

## Discussion

The present study is the first to provide a detailed spatiotemporal relationship between astrocytes and OPN puncta, which indeed corresponded to degenerating neurites in the lesioned striatum following 3-NP injection. OPN puncta were localized away from reactive astrocytes at 3 days post-lesion, but the distance between OPN puncta and the astroglial scar progressively closed following the injury time course, and eventually, at 28 days, their gross distribution overlapped. In particular, OPN puncta were closely associated with, or completely engulfed by, reactive astrocytes at 14–28 days. This was confirmed by ultrastructural investigation using immunoelectron microscopy. Thus, our data support the recent proposal that OPN is involved in the activation and polarization of astrocytes after brain injury [14, 27], and provide evidence that astrocytes are likely to respond to OPN-labeled degenerating neurites, and subsequently phagocytose them after brain insults.

Astrocytic scar formation involves the transition of quiescent astrocytes into hypertrophied reactive astrocytes, astrocytic process elongation, polarization of their processes, and migration into the lesion core [14, 46–48]. Several studies have documented the possibility that OPN is involved in cellular migration by its interaction with two receptors for OPN, α_v_β_3_ integrin and CD44 [17, 49–53]. In particular, Zohar et al. demonstrated that OPN and CD44 are expressed in the leading edge of migrating fibroblasts and mediate cell migration through interaction with ezrin-radixin-moesin protein [50]. In addition, OPN has been shown to enhance the migration of human monocytes and modulate microglia motility with involvement of the FAK-ERK-AKT pathway via integrin receptor binding [53, 54]. Moreover, previous studies have elucidated the concomitant induction of OPN and CD44 or integrin receptors in reactive astrocytes after various CNS injuries [4, 55–58]. The chemotactic role of OPN and CD44-mediated migration has been especially demonstrated in C6 astroglioma and primary astrocytes [17, 57]. In line with this, we speculate that at 3 days after the injury, punctate OPN produced by amoeboid brain macrophages in the lesion core act as a chemoattractant, facilitating astrocyte migration toward the lesion core, thereby decreasing the distance between the OPN-positive lesion core and astrocytic scar processes at day 7 (Fig. 5).

In addition to the role of OPN in cellular migration, recent studies have shown that astrocyte polarization is linked to the integrin subunit and that depletion of hematogenous OPN resulted in the defective polarization of reactive astrocytes in a photothrombic stroke model [14, 59]. Furthermore, OPN deficient mice exhibited attenuation of reactive astrogliosis after stab wound injury [27], while the absence of OPN in the retina displayed a deficit in astrocyte coverage and linear extension and process branching indicative of premature aging [28]. In this regard, OPN’s overlapping distribution and often direct contact with astrocytes at days 7–14 after the 3-NP injury indicate a probable direct interaction of OPN protein with scar-forming astroglia, possibly involving the modulation of process elongation and polarization. In support of this, delayed upregulation of OPN and integrin receptor α_v_β_3_ within reactive astrocytes after cerebral ischemia temporally correlates with astrocytic scar formation, supporting the possible role of OPN in such processes [4, 17]. However, further study is needed to clarify the signaling mechanisms underlying this process.

Furthermore, we demonstrated that numerous OPN puncta were localized within the cytoplasm of reactive astrocytes at 28 days after 3-NP treatment and that they were indeed degenerated neurites with shrunken, electron-dense mitochondria inside, as verified by electron microscopy. In addition, triple-labeling of OPN, GFAP, and lysosomal marker LAMP1 clearly revealed that reactive astrocytes including OPN puncta contained numerous lysosomes, implying their functionally phagocytic activity. Moreover, OPN puncta engulfed by reactive astrocytes exceeded those of activated microglia/macrophages within the astroglial scar throughout the post-injury time frame (7, 14, and 28 days). Thus, our data indicate that reactive astrocytes forming the astroglial scar are capable of phagocytosing OPN puncta in the lesioned striatum. Interestingly, OPN protein accumulates selectively on the surface of degenerating neurites that are filled with aggregated calcium crystals [8, 10, 16], and the distribution of the calcifying deposits was frequently observed in close proximity to, surrounded by, or sometimes within, hypertrophied reactive astrocytes, suggesting possible phagocytosis of calcium deposits by reactive astrocytes [9, 60, 61]. Therefore, it is plausible to conclude that degenerative calcifying neurites labeled by OPN were often phagocytosed by reactive astrocytes.

In addition to microglia and infiltrating blood-borne macrophages, which are considered to be professional phagocytes in the brain, accumulating evidence suggests an active astrocyte role in phagocytosis under various circumstances [62–66]. During development, astrocytes have been shown to phagocytose synapses involving the MEGF10-MERTK pathways [63]. Additionally, in vitro astrocytes were able to effectively engulf dead cells after neural scratch injury [62]. Furthermore, dystrophic neurites in Alzheimer’s were phagocytosed by reactive astrocytes, but not by microglia [66]. In particular, Morizawa et al. elucidated the increase in ABCA1-mediated phagocytosis of degenerating neuronal debris by reactive astrocytes in ischemic stroke [65]. In this study, phagocytosis by microglia predominated in the lesion core in the acute phase, while astrocytic phagocytosis was more evident in the penumbra region in the subacute phase, corroborating our findings where the astrocytic phagocytosis of OPN puncta peaked at 14–28 days post-injury in the peri-lesional astroglial scar area.

Countless studies have suggested the immuno-modulatory role of OPN in brain pathologies [13, 25, 67–70]. Particularly, OPN has been shown to enhance the phagocytosis of monocytes and microglia [52–54]. OPN binds to bacteria in a specific manner and opsonizes them for phagocytosis via the integrin receptor [54], while one in vitro study demonstrated that administration of OPN enhanced the phagocytic activity of microglia via the FAK, Erk, and Akt signaling pathways [53]. In addition, we have previously suggested the phagocytosis of OPN-coated degenerative neurites by brain macrophages in ischemic stroke [8]. However, the present work is the first to elucidate the direct involvement of OPN in the phagocytosis of reactive astrocytes, in addition to brain macrophages.

Recent transcriptomic studies have revealed that cerebral ischemia and neuroinflammation differentially regulate astrocyte reactivity and categorized reactive astrocytes into two types: A1 (neurotoxic) and A2 (neuroprotective) astrocytes. A1 astrocytes, which predominate after neuroinflammation, are deemed harmful due to the upregulation of pro-inflammatory genes and a deficiency in phagocytic ability, while A2 neuroprotective astrocytes, prevailing after ischemic stroke, are characterized by the expression of neurotrophic factors and cytokines including a gene encoding OPN [71–73]. Interestingly, administration of exogenous OPN conferred neuroprotective effects in ischemia, and OPN-deficient mice exhibited aggravated lesion development after stroke and spinal cord injury [12, 13, 73–78]. Thus, OPN-mediated astrocytic phagocytosis after acute focal CNS injury may involve the neuroprotective response of A2 reactive astrocytes, yet future studies are required to elucidate the detailed mechanism by which this occurs.

## Conclusions

In conclusion, we demonstrated that OPN puncta corresponding to degenerated neurites surrounded by the OPN protein that was synthesized and secreted by brain macrophages became closely associated with, or completely engulfed by, reactive astrocytes forming the astroglial scar in 3-NP lesioned striatum. Our data suggest that OPN may cause astrocytes to migrate toward these degenerated neurites in the lesion core, to establish physical contact with, and possibly, phagocytose them. Thus, our results provide novel insights into the role of OPN in glial scar formation and astrocytic phagocytosis after CNS injury, which are essential to understanding the recovery and repair of CNS tissue.

## Additional files

Additional file 1: **Figure S1.** Overlapping distribution of OPN-immunoreactive profiles using two kinds of antibodies against OPN. (A–F) Note that two OPN antibodies, i.e., the mouse monoclonal and goat polyclonal antibodies, have an overlapping distribution in the lesioned striatum at 7 days post-lesion, and OPN-positive staining is visible as small granular puncta. The boxed areas in A–C are enlarged in D–F, respectively. Cell nuclei appear blue after DAPI staining. Scale bars = 50 μm for A–C and 10 μm for D-F. (TIF 8632 kb)

Additional file 2: **Figure S2.** The presence of lysosomal marker LAMP1 within activated microglia/macrophages. (A–C) Double labeling with Iba1 and LAMP1 in the lesioned striatum at 28 days post-lesion, showing that prominent LAMP1 staining (red arrows) with a typical punctate pattern is localized within Iba1-positive activated microglia/macrophages. Note the presence of LAMP1 staining (yellow arrowheads) outside the Iba1-positive cells. Cell nuclei appear blue after DAPI staining. Scale bars = 10 μm for A–C. (TIF 3640 kb)

## Abbreviations

3-NP: 3-Nitropropionic acid
CNS: Central nervous system
GFAP: Glial fibrillary acidic protein
Iba1: Ionized calcium-biding adaptor molecule 1
LAMP1: Lysosomal-associated membrane protein 1
OPN: Osteopontin
SPP1: Secreted phosphoprotein 1
