# Supplementary figures and images for: Osteopontin and its spatiotemporal relationship with glial cells in the striatum of rats treated with mitochondrial toxin 3-nitropropionic acid: possible involvement in phagocytosis
Source: J Neuroinflammation. 2019 May 14;16:99. doi: 10.1186/s12974-019-1489-1 (PMC6518780; doi:10.1186/s12974-019-1489-1)

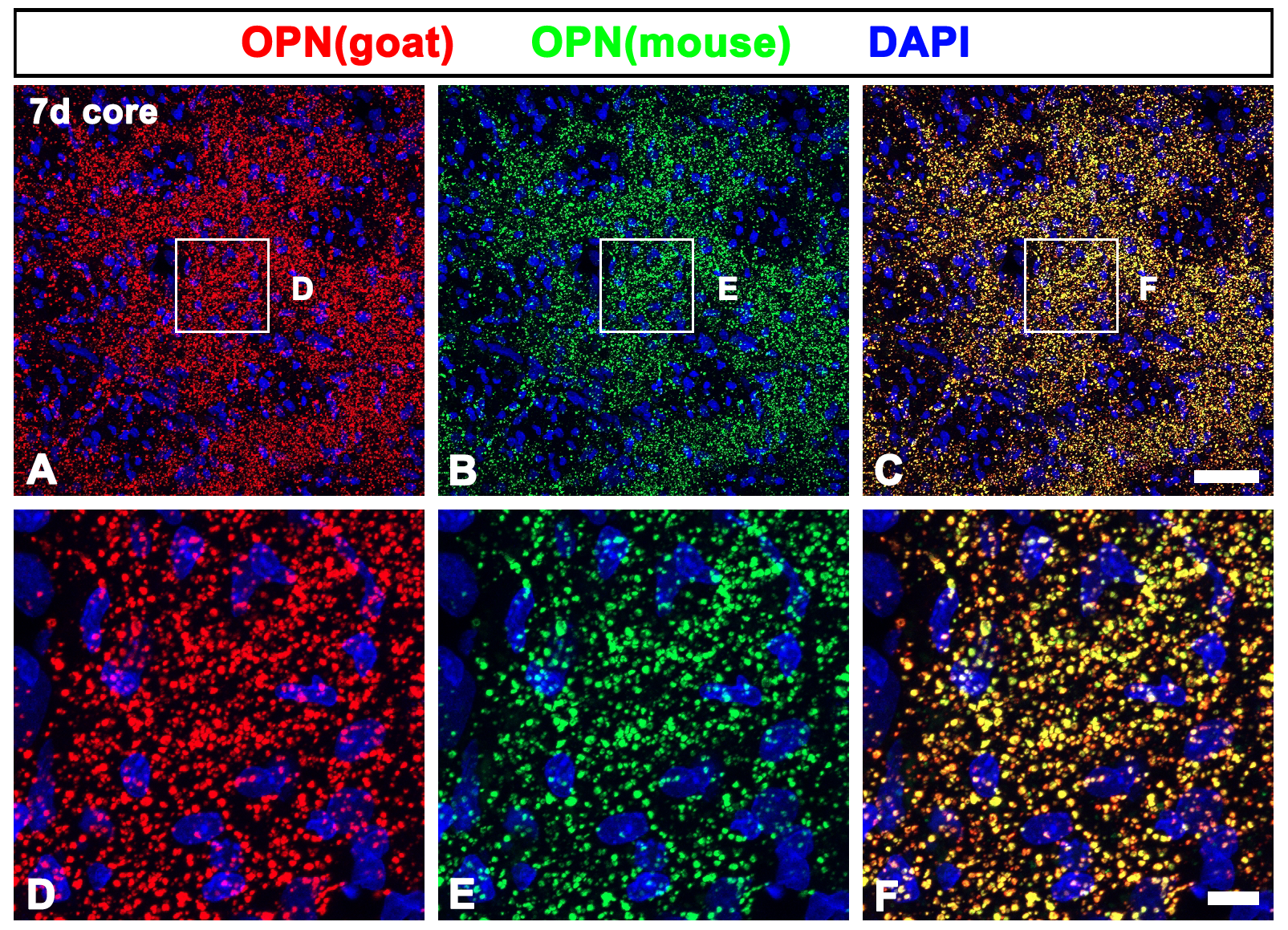

Supplement: Supplementary file 1 — Figure S1. Overlapping distribution of OPN-immunoreactive profiles using two kinds of antibodies against OPN. (A–F) Note that two OPN antibodies, i.e., the mouse monoclonal and goat polyclonal antibodies, have an overlapping distribution in the lesioned striatum at 7 days post-lesion, and OPN-positive staining is visible as small granular puncta. The boxed areas in A–C are enlarged in D–F, respectively. Cell nuclei appear blue after DAPI staining. Scale bars = 50 μm for A–C and 10 μm for D-F. (TIF 8632 kb) [file 12974_2019_1489_MOESM1_ESM.tif]

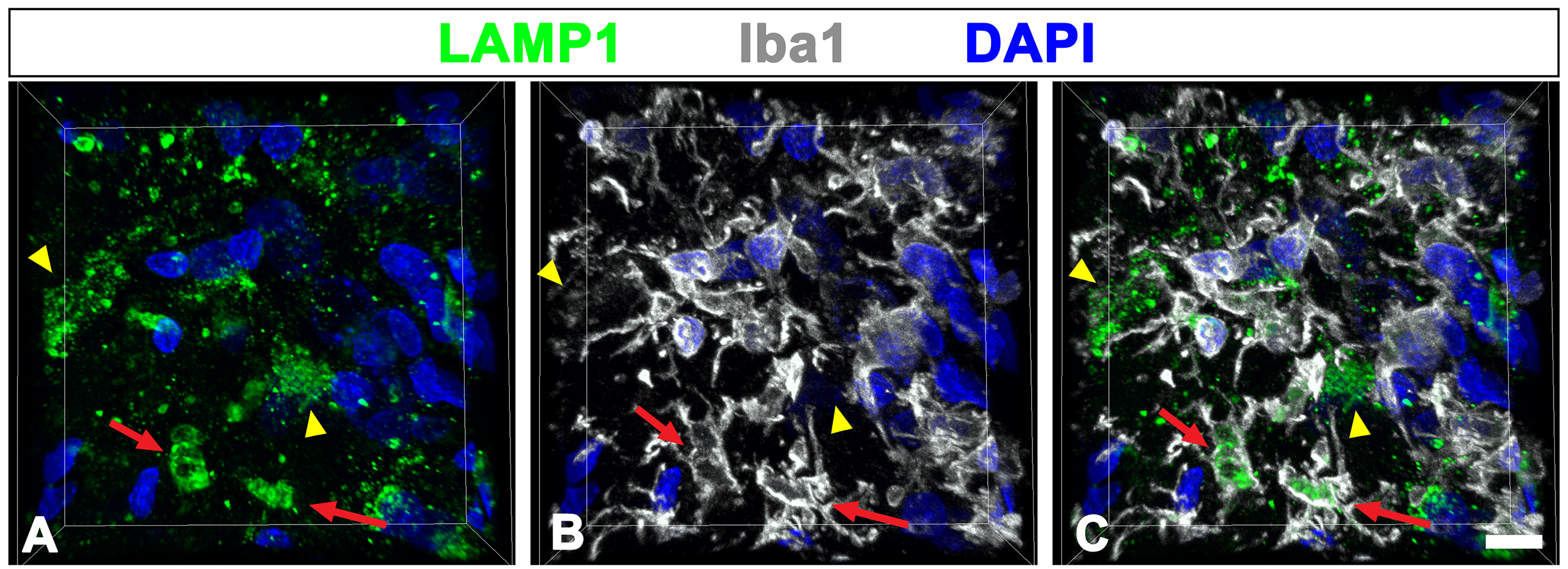

Supplement: Supplementary file 2 — Figure S2. The presence of lysosomal marker LAMP1 within activated microglia/macrophages. (A–C) Double labeling with Iba1 and LAMP1 in the lesioned striatum at 28 days post-lesion, showing that prominent LAMP1 staining (red arrows) with a typical punctate pattern is localized within Iba1-positive activated microglia/macrophages. Note the presence of LAMP1 staining (yellow arrowheads) outside the Iba1-positive cells. Cell nuclei appear blue after DAPI staining. Scale bars = 10 μm for A–C. (TIF 3640 kb) [file 12974_2019_1489_MOESM2_ESM.tif]
